# Editorial: Motivation-based approaches to countering mass-mediated misinformation

**DOI:** 10.3389/fpsyg.2024.1501995

**Published:** 2024-10-10

**Authors:** Claude H. Miller, Elena Bessarabova, Bobi Ivanov, John A. Banas

**Affiliations:** ^1^Department of Communication, University of Oklahoma, Norman, OK, United States; ^2^College of Communication and Information, University of Kentucky, Lexington, KY, United States

**Keywords:** bias, disinformation, framing, inoculation, misinformation, motivation, pre-bunking, resistance to influence

In the contemporary information ecosystem, misinformation and disinformation pose significant challenges to public understanding and the integrity of democratic processes. Addressing these challenges requires both comprehending the nature of false information as well as the psychological underpinnings that make individuals susceptible to it. The articles included in this Research Topic collectively explore motivation-based strategies as a means of counteracting the dissemination and spread of misinformation and disinformation, emphasizing the psychological mechanisms that drive belief in false information and how they can be leveraged to enhance resilience against such threats. Each article addresses different facets of motivation and its impact on misinformation processing, yet they converge on the central theme that motivation is a powerful force in shaping individuals' receptivity to information and their ability to discern truths from falsehoods. The studies delve into ideological, cultural, emotional, and cognitive motivations, demonstrating how these can either facilitate or hinder the acceptance of misinformation and disinformation.

In “*Ideological predictors of anti-science attitudes: exploring the impact of group-based dominance and populism in North America and Western Europe*,” Remsö and Renström highlight how ideological motivations, such as group-based dominance, populism, symbolic ideology, and scientific literacy can foster skepticism toward science. Although the effect of populism tends to vary, their study finds group-based dominance to be a strong predictor of anti-science attitudes. Remsö and Renström's work underlines how deeply ingrained beliefs can lead to the rejection of scientific facts and stresses how addressing these ideological roots is crucial in efforts to counter the formation and spread of misinformation.

Harmon-Jones et al., in “*Evil perceptions but not entertainment value appraisals relate to conspiracy beliefs*,” hypothesize that, rather than entertainment value, the perception of evil intentions by conspirators mediates beliefs in conspiracy theories and find that such perceptions play a key role in supporting implausible beliefs in the absence of credible evidence. The researchers note how emotional motivations, particularly those rooted in moral judgments, appear to be significant predictors of misinformation acceptance and stress how addressing the emotional and moral appraisals associated with perceptions of evil can play a central role in reducing belief in conspiracy theories.

In “*What motivates bridge building across pernicious group divides*? *The effects of regulatory motives, framing, and fit on increasing constructive engagement across political and racial divisions*,” Coleman and Phan hypothesize how regulatory motives such as prevention and promotion, along with framing and motivational fit, can affect engagement in bridge-building activities between antagonistic outgroups. Their work on the factors motivating social cohesion examines how various motivational strategies can facilitate dialogue across political and racial divides. Their findings point to how such strategies can cultivate conditions favorable for factual accuracy, which in turn helps mitigate the spread of misinformation. Coleman and Phan highlight the potential of motivation-based interventions to reduce polarization, aid in more critical engagement with information, and thereby indirectly counter false, misleading, and erroneous information.

Bessarabova et al., in “*Assessing inoculation's effectiveness in motivating resistance to conspiracy propaganda in Finnish and United States samples*,” demonstrate the efficacy of inoculation strategies for building resistance to conspiracy theory misinformation. The authors show how preemptively addressing the motivational threats posed by false information can enhance individuals' analytic thinking. Their results indicate that, although intuitive thinking can be positively associated with disinformation endorsement, analytic engagement with inoculation materials can effectively reduce such validation. Taken together, their data suggest inoculation-based pre-bunking strategies can be effective across different cultures to provide a viable means of mitigating the expanding threat of misleading narratives, factual distortions, and deliberate deceptions associated with conspiracy theories.

In “*Using a signal detection approach to understand the impacts of processing fluency and efficacy on accuracy in misinformation detection*,” Fort and Shulman provide valuable insights into the cognitive processes that influence how individuals evaluate and identify false information by delving into the intricate relationship between processing fluency, internal efficacy, and the ability to detect misinformation. Hypothesizing how the two constructs can influence the accuracy of misinformation detection, they find that a state of metacognitive ease enhances individuals' confidence and accuracy in detecting misinformation, highlighting the role of internal efficacy as a motivational factor in promoting critical evaluation of information. By emphasizing the roles of processing fluency and internal efficacy, their work opens up new avenues for developing more effective strategies for combating the spread of disinformation in our increasingly complex information landscape.

Finally, in “*Processing of misinformation as motivational and cognitive biases*,” Zhou and Shen provide a review of the motivational and cognitive biases contributing to the persistence of misinformation, exploring how non-accuracy motivations and different cognitive biases can contribute to misinformation persistence. As do Bessarabova et al., Zhou and Shen advocate the use of inoculation as a prebunking strategy, and emphasize the need for addressing the underlying psychological factors that sustain misinformation by preparing individuals to critically evaluate the nature of the information they consume so as to counteract their biases before they are exposed to misleading content.

Overall, these articles underscore the importance of understanding and leveraging motivational factors crucial for developing effective misinformation countermeasures. By addressing psychological mechanisms driving belief in false information, the motivation-based approaches examined offer promising avenues for fostering skepticism and enhancing public resilience against mass-mediated misinformation and disinformation. The articles emphasize how interventions should target ideological motivations, emotional appraisals, and cognitive biases to enhance resilience against disinformation supporting conspiracy theories and fabricated content. Strategies such as inoculation and framing offer promising avenues for addressing psychological roots of misinformation acceptance.

The consensus across these studies is that understanding and leveraging motivational factors is essential for developing interventions to counter so-called “fake news,” promote informed decision-making, and safeguard public discourse. By incorporating advances in new technologies and employing interdisciplinary approaches, future research should continue to examine motivational interventions aimed at countering deception, mitigating bias, and thwarting the creation and spread of misinformation.

